# MRI assessment of aortic flow and pulse wave velocity in response to exercise

**DOI:** 10.1186/1532-429X-17-S1-M2

**Published:** 2015-02-03

**Authors:** Jacob Macdonald, Omid Forouzan, Jared Warczytowa, Oliver Wieben, Christopher J Francois, Naomi C Chesler

**Affiliations:** 1 Medical Physics, University of Wisconsin, Madison, WI, USA; 2 Biomedical Engineering, University of Wisconsin, Madison, WI, USA; 3 Radiology, University of Wisconsin, Madison, WI, USA

## Background

The effect of exercise on cardiac function is important in the diagnosis of cardiovascular disease [1], yet is clinically not available with MRI. Instead, pharmacological agents are used, which have several disadvantages [2]. Recent work has addressed this shortcoming with an MR compatible ergometer next to the magnet [3], albeit with an unavoidable time delay between exercising and actual scanning, which can reduce the measured effect. In this pilot study, we investigated flow and area changes in the aorta with customized exercise equipment that functions in the bore.

## Methods

**Figure 1:**
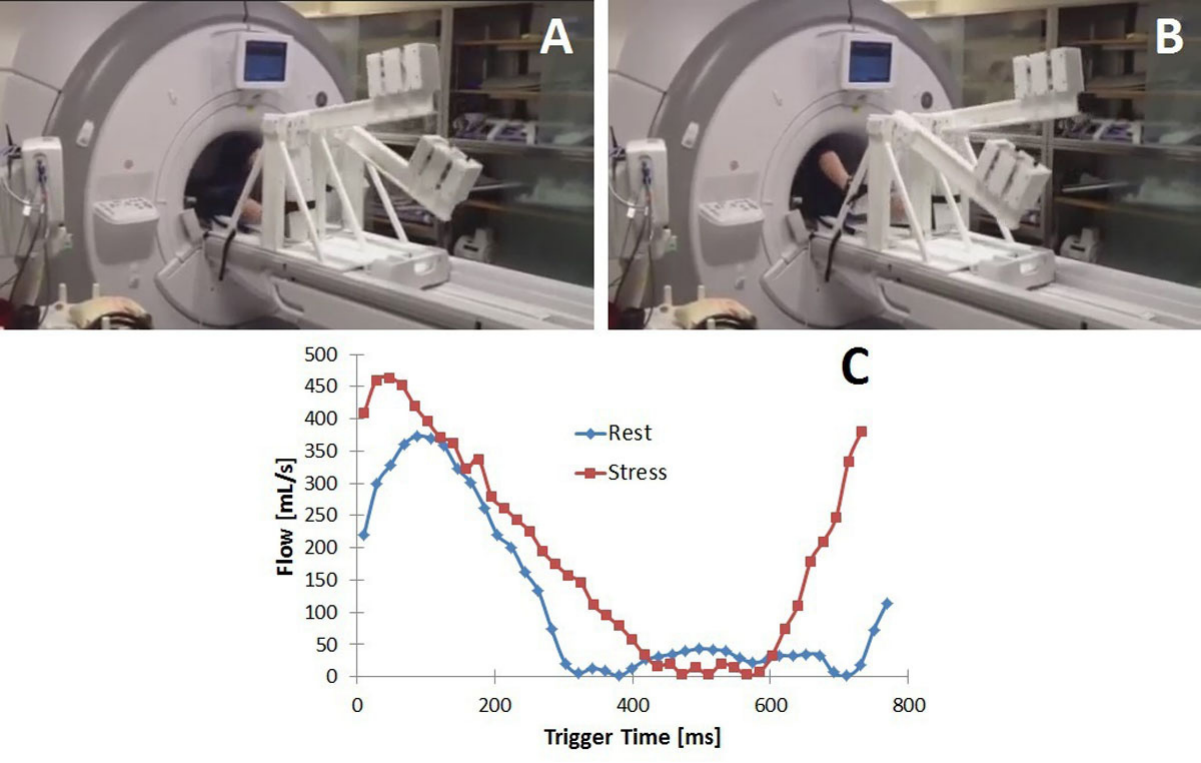
MRI-compatible stepper exercise device. The subject extends and then flexes alternating knees in a dynamic stepping motion as shown in (A) and (B). The resistance is controlled by weights on the lever. Subjects step to the beat of a metronome, and the workload is calculated with readings from an optical displacement sensor on the levers and the frequency of the motion. The device allows comparison of parameters in the same scan plane under rest and stress conditions, as shown in (C).

Twelve healthy volunteers were imaged on a clinical 1.5T system (HDx (Ø=60cm) and 450w (Ø=70cm), GE Healthcare). A custom-made MR-compatible stepping device was used which allowed subjects to exercise in a supine position (Figure 1) [4]. The subjects exercised in 3 successive, incremental exercise stages with workloads of 36±7, 43±6, and 50±8 W. Each exercise stage was 3 minutes long and followed immediately by a gated 2D cine PC acquisition (TR/TE=6.1/3.7s; FA=30º; ASSET=2; VENC=150cm/s) in the ascending aorta across a 15s breath hold. The subject's heart rate was recorded during the scan. Changes in peak systolic velocity, peak systolic flow, cardiac output, relative aorta area change, heart rate, and pulse wave velocity (using the QA method [5]) were analyzed for each exercise stage. A two-sample, dependent t-test was used to determine the statistical significance of any changes between exercise and baseline. Here, results are presented for the second exercise stage, as it had the smallest intrasubject variation in work load.

## Results

**Figure 2:**
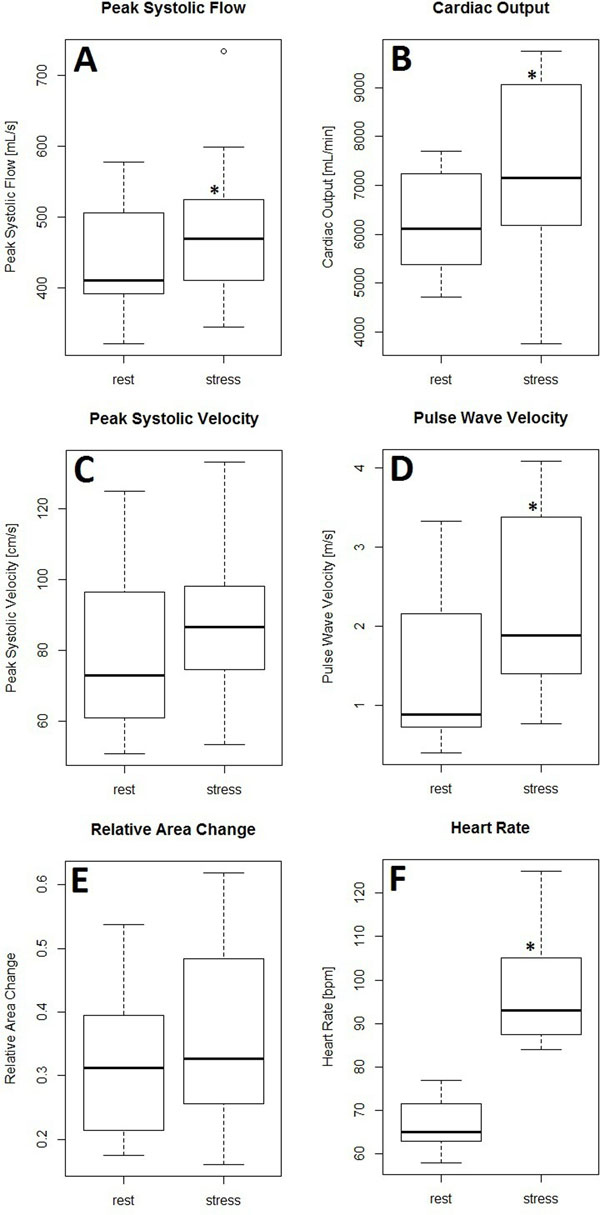
Measurements of (A) peak systolic flow, (B) cardiac output, (C) peak systolic velocity, (D) pulse wave velocity, (E) relative area change, (F) heart rate. An asterisk represents a statistically significant (p < 0.05) increase of the parameter under exercise conditions when compared to the baseline.

Figure 2 summarizes the results. Peak systolic velocity, cardiac output, pulse wave velocity, and heart rate had statistically significant increases. The increase in heart rate validated that the exercise equipment was effective at inducing moderate exercise stress for the scan duration. Peak systolic velocity, cardiac output, and pulse wave velocity had mean increases of 10%, 76%, and 67% respectively. This suggests cardiac output and pulse wave velocity are most sensitive to changes in flow dynamics. Several datasets from the third exercise stage (not shown here) were more affected by motion artifacts, presumably due to fatigue of the subjects from repeated and increased exercise loads.

## Conclusions

This feasibility study demonstrates the use of a customized exercise device that allows for aortic flow measurements that characterize the effect of exercise stress without position changes. Cardiac output and pulse wave velocity demonstrated the greatest sensitivity to exercise stress. In future studies, we will investigate the diagnostic value of these hemodynamic parameters in patient populations, specifically in subjects with diastolic dysfunction.

## Funding

NIH R01 HL105598.
